# Supplementary material for: Abdominal aortic aneurysm and cardiometabolic traits share strong genetic susceptibility to lipid metabolism and inflammation
Source: Nat Commun. 2024 Jul 5;15:5652. doi: 10.1038/s41467-024-49921-7 (PMC11226445; doi:10.1038/s41467-024-49921-7)
Supplement: Supplementary file 1 — Supplementary Information [file 41467_2024_49921_MOESM1_ESM.pdf]

Abdominal Aortic Aneurysm and Cardiometabolic Traits Share Strong Genetic Susceptibility to Lipid Metabolism and Inflammation

Shufen Zheng[1,2], Philip S. Tsao[3,4,5*], Cuiping Pan[1,2*]

1. Center for Intelligent Medicine Research, Greater Bay Area Institute of Precision Medicine (Guangzhou), Guangzhou, China
2. Center for Evolutionary Biology, Intelligent Medicine Institute, School of Life Sciences, Fudan University, Shanghai, China.
3. Division of Cardiovascular Medicine, Stanford University School of Medicine, Stanford California, USA
4. Stanford Cardiovascular Institute, Stanford University, California, USA
5. VA Palo Alto Health Care System, Palo Alto, California, USA

Supplementary Figures

Supplementary Figure 1. Partitioned genetic correlation between AAA and CMTs.

Supplementary Figure 2. Number of shared SNVs between AAA and CMTs via MTAG and CPASSOC.

Supplementary Figure 3. Number of genes for each trait pair identified by four methods: GCTA, MAGMA, TWAS, and SMR.

Supplementary Figure 4. Genes identified by all four gene-centric analysis methods and shared by minimally three AAA trait pairs.

Supplementary Figure 5. GO and KEGG pathway enrichment analysis for AAA and CMTs.

Supplementary Figure 6. Tissue type enrichment of the shared signals between AAA and CMTs.

Supplementary Figure 7. Cell type enrichment of the shared signals between AAA and CMTs.

Supplementary Figure 8. Direction of effect by the genes on the CMTs in tissue context.

Supplementary Figure 9. Interpretation of AAA-associated variants identified by GWAS – variants, genes, tissues and cell types.

Supplementary Figure 10. Interpretation of AAA-associated variants identified by GWAS – Biological pathways and drugs.

(Source data are provided with this paper)

Supplementary Tables

Supplementary Table 1. Horizontal pleiotropy analyses between AAA and cardiometabolic traits by MR-Egger.

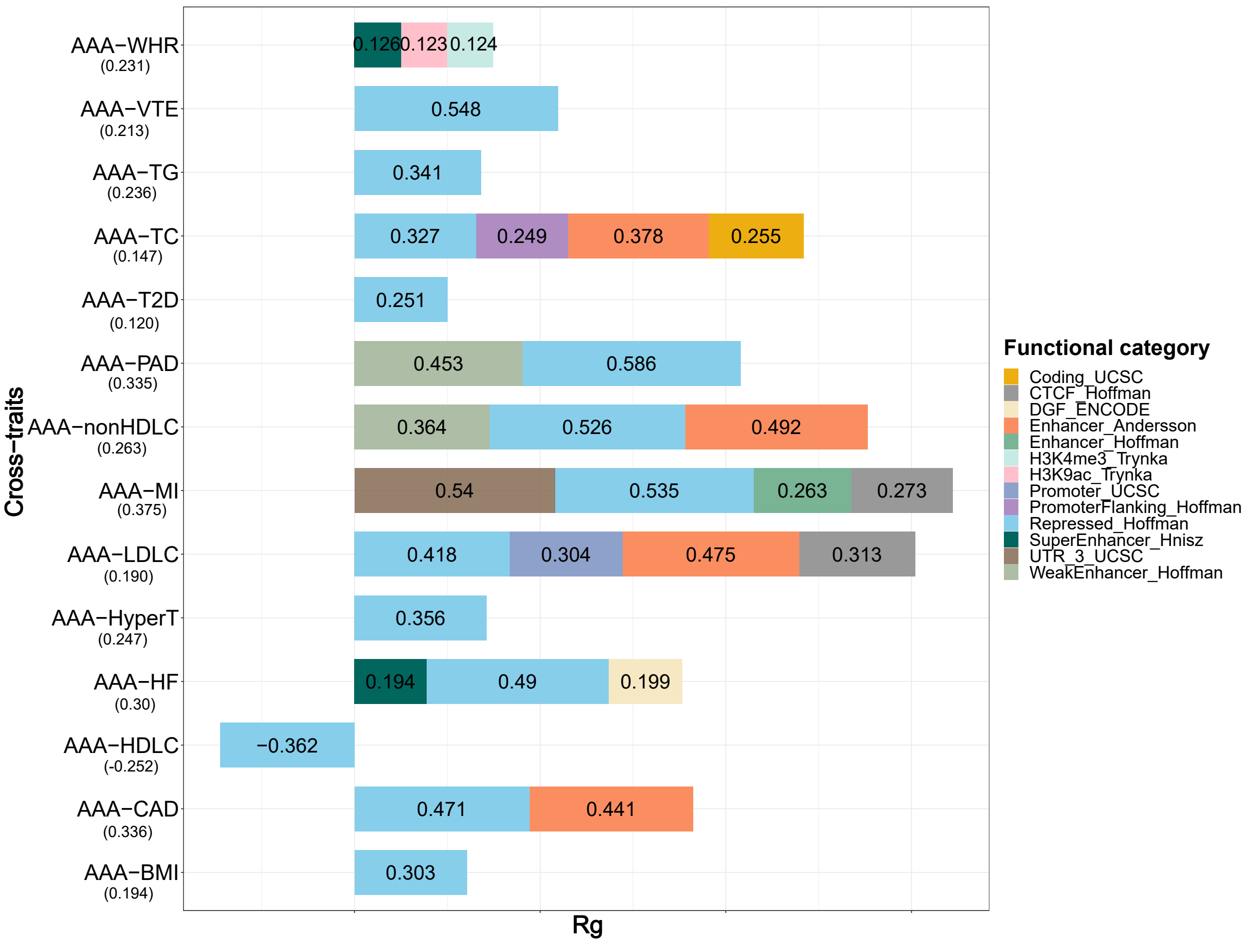

Supplementary Figure 1. Partitioned genetic correlation between AAA and CMTs. 24 types of genomic functional elements were interrogated, as labeled on the left. The genome-wide correlation values were depicted in parenthesis under each trait pair. The bar plot shows functional categories of different correlation than the genome-wide correlation, as defined by a difference rg ≥ |0.1| to the genome-wide correlation value. Colors represent different functional categories.

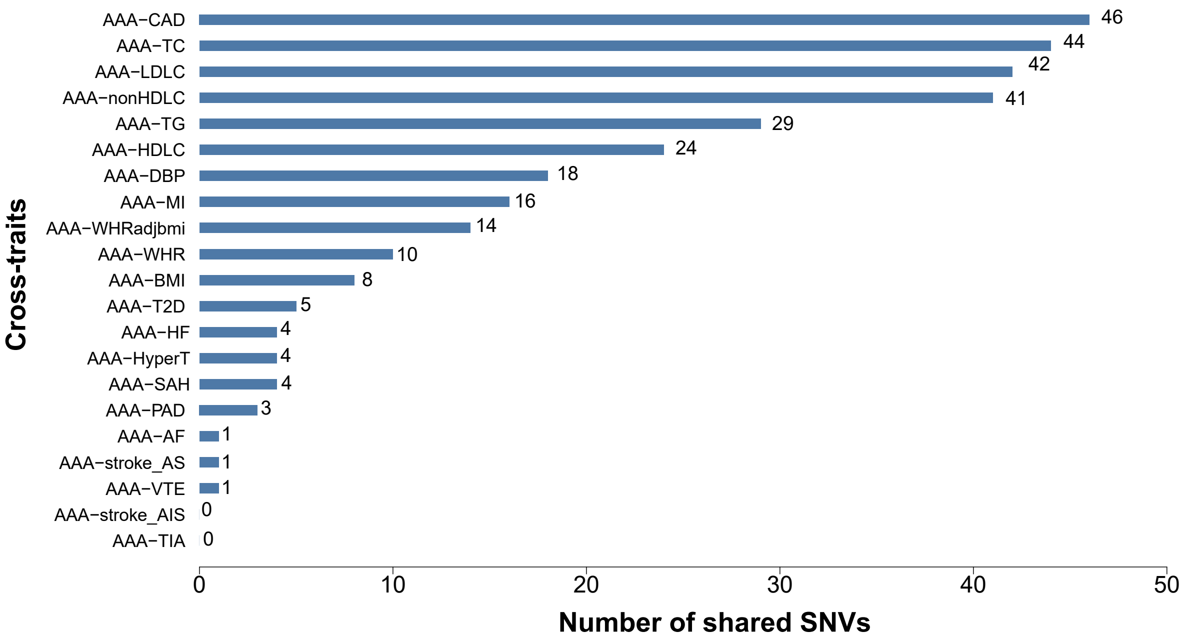

Supplementary Figure 2. Number of shared SNVs between AAA and CMTs via MTAG and CPASSOC.

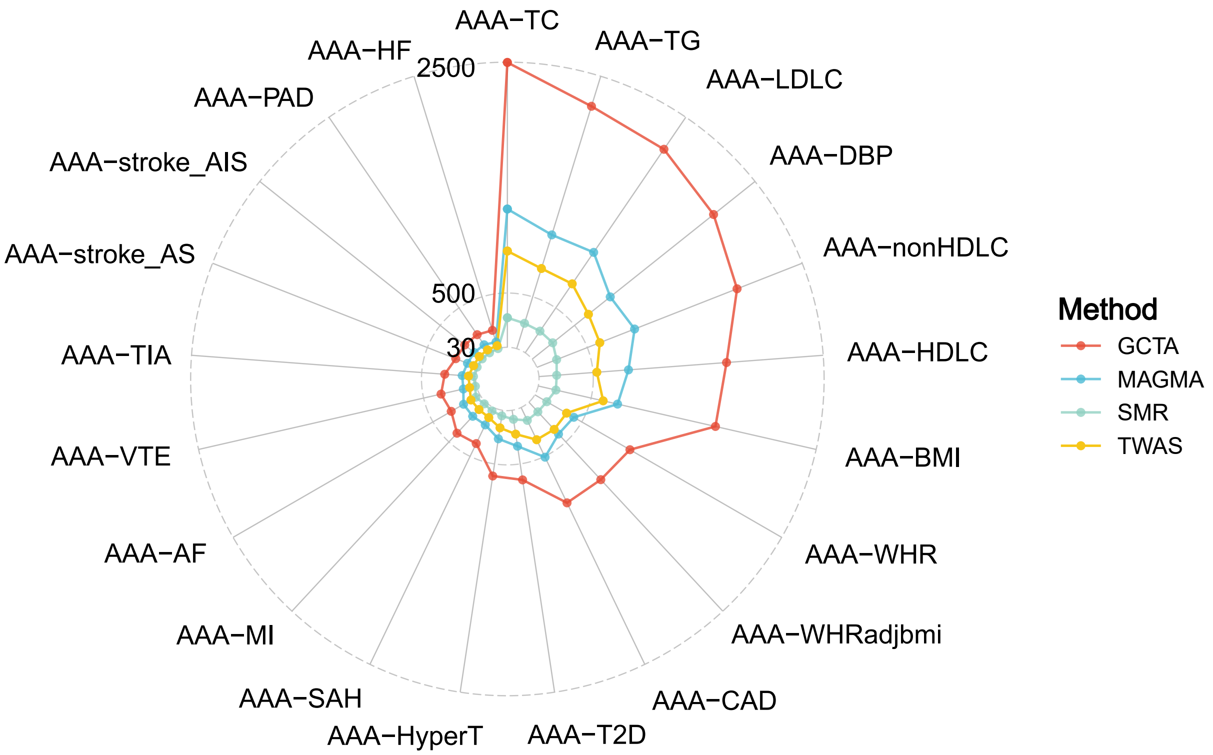

Supplementary Figure 3. Number of genes for each trait pair identified by four methods: GCTA, MAGMA, TWAS, and SMR. Each method is represented by one color. The numbers of identified genes are marked on each tier. Bonferroni correction was applied to adjust for multiple testing.

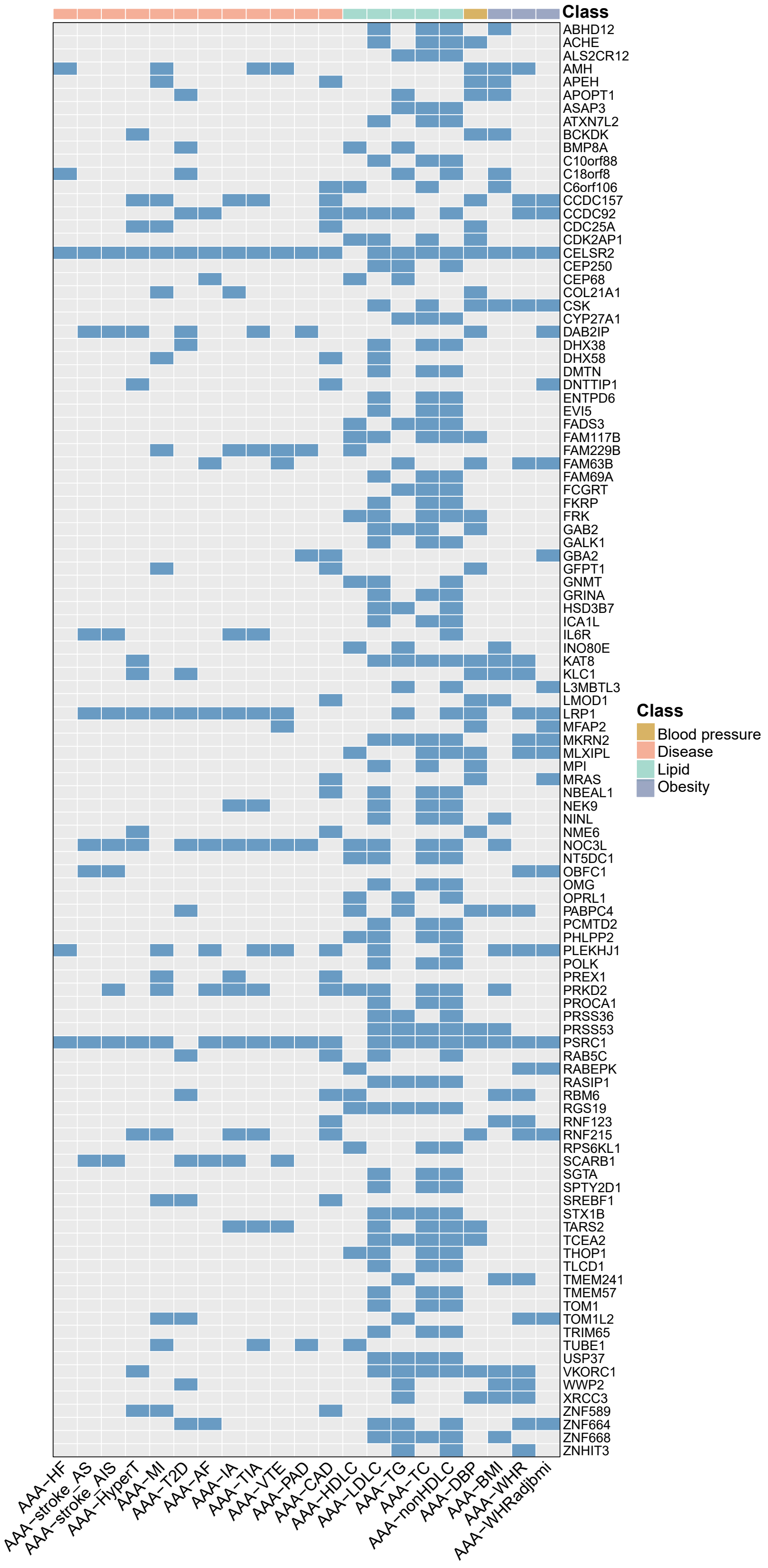

Supplementary Figure 4. Genes identified by all four gene-centric analysis methods and shared by minimally three AAA trait pairs. The four gene analysis methods are: GCTA, MAGMA, TWAS, and SMR. Genes are labeled on the bottom, and the trait pairs are labeled on the right. Blue color indicates presence of the gene. For each analysis, we corrected for multiple testing using Bonferroni correction.

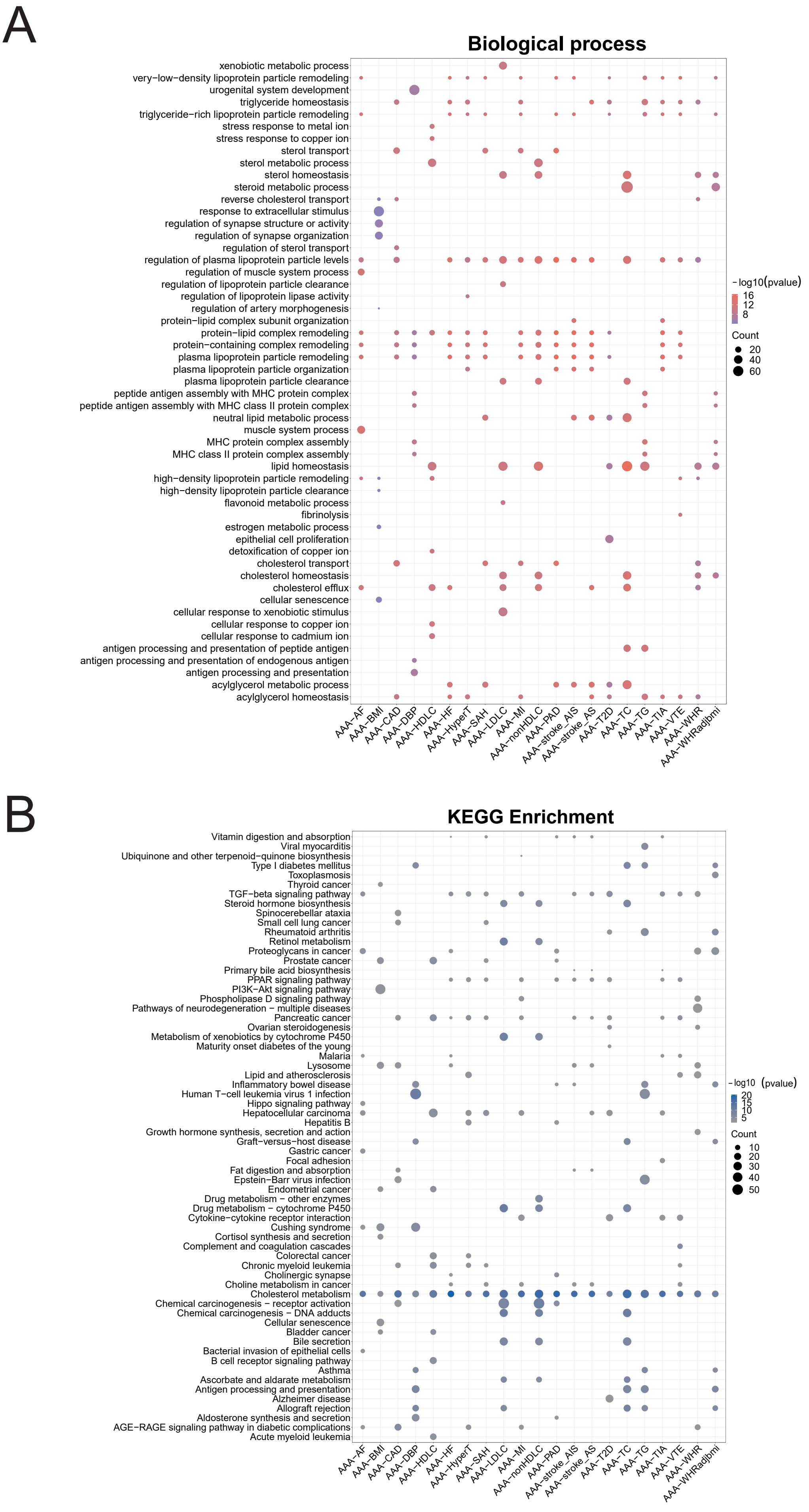

Supplementary Figure 5. GO and KEGG pathway enrichment analysis for AAA and CMTs. A. GO term (biological process) enrichment. B. KEGG pathway enrichment. Genes were derived from the union of four gene analysis methods: GCTA, MAGMA, TWAS, and SMR. FDR was calculated via the Benjamin–Hochberg method to correct for multiple testing. The top 10 enriched pathways with FDR-adjusted P < 0.05 in each trait pair were included.

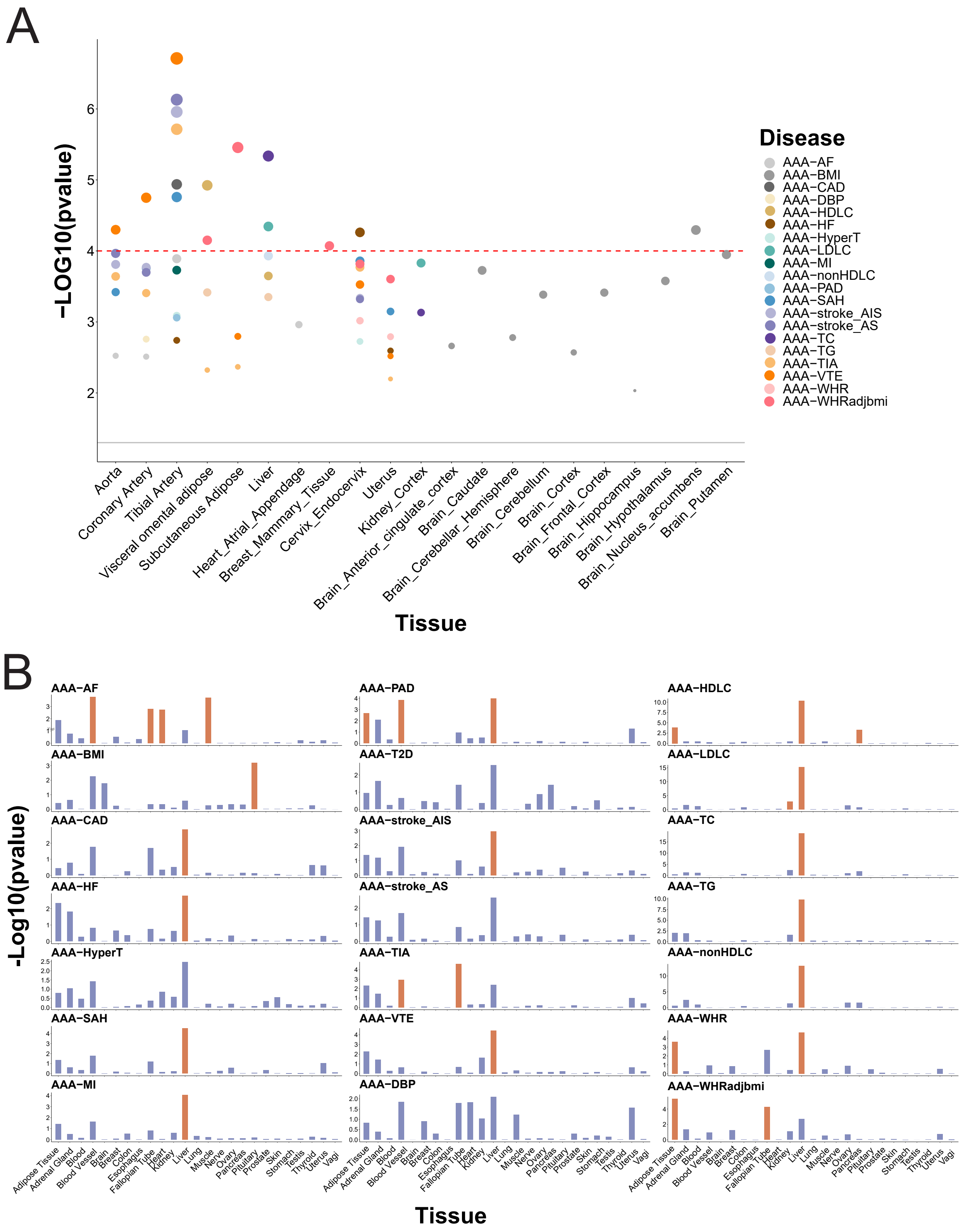

Supplementary Figure 6. Tissue type enrichment of the shared signals between AAA and CMTs. A. Enriched by heritability of the tissue-specific genes derived from GTEx. The red dashed line represents P < 0.0001, an arbitrarily defined threshold. B. Enriched by the tissue-specific expression in GTEx. Brown bars represent significant enrichment, i.e., passing the Bonferroni-corrected P value threshold, whereas blue bars indicate not passing the Bonferroni correction.

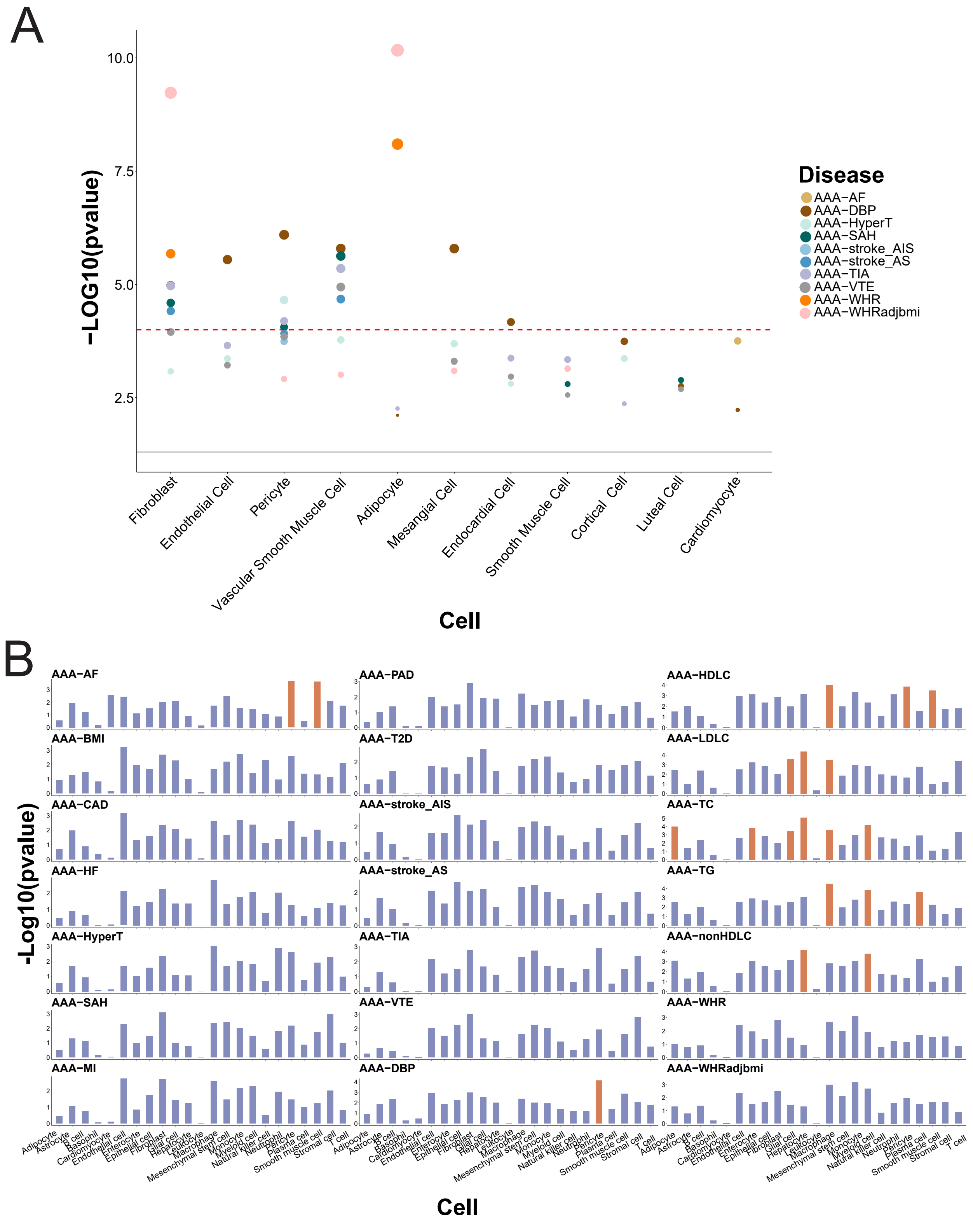

Supplementary Figure 7. Cell type enrichment of the shared signals between AAA and CMTs. A. Enriched by heritability of the cell type-specific enhancers derived from CATLAS. The red dashed line represents P < 0.0001, an arbitrarily defined threshold. B. Enrichment by cell type-specific expression by referencing to 11 single-cell transcriptome datasets. Brown bars represent significant enrichment, i.e., passing the Bonferroni-corrected P value threshold, whereas blue bars indicate not passing the Bonferroni correction.

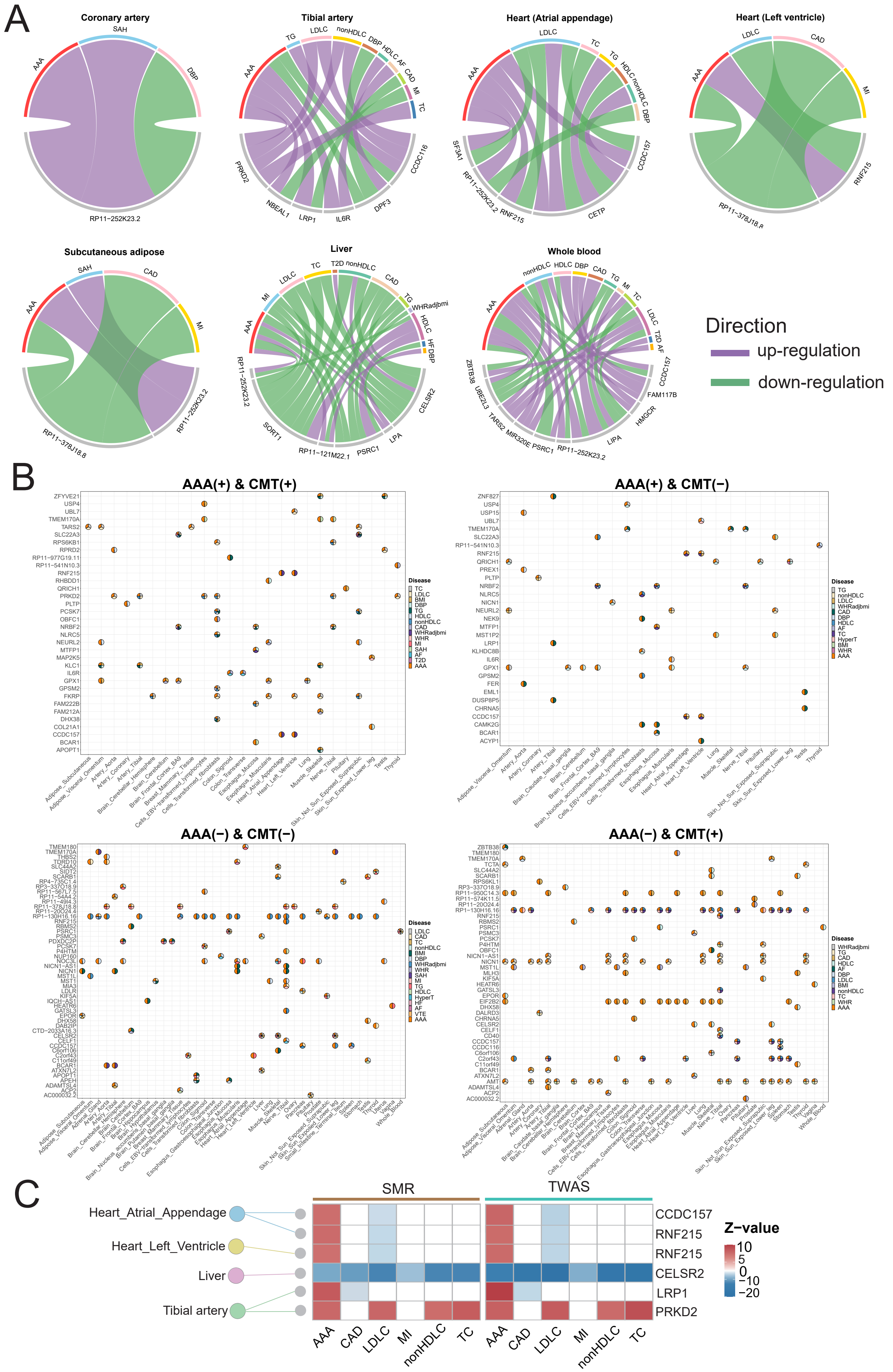

Supplementary Figure 8. Direction of effect by the genes on the CMTs in tissue context. A. SMR-derived directions of effect, inferred by the top causal eQTLs. Traits are marked in the upper half circles, and the genes are marked in the lower half circles. Lines within the circles connect genes and the traits. The line color indicates positive (purple) or negative (green) correlation, and the width indicates strength of association derived from Z-scores. Nine tissues impacted the most by the shared signals between AAA and CMTs were examined. B. Fusion-derived directions of effect, utilizing the model of cis-SNVs on gene expression. 49 tissues from GTEx were interrogated. C. Overlapped results with cross trait - tissue - gene connections identified by both SMR and TWAS. Bonferroni correction was applied to adjust for multiple testing.

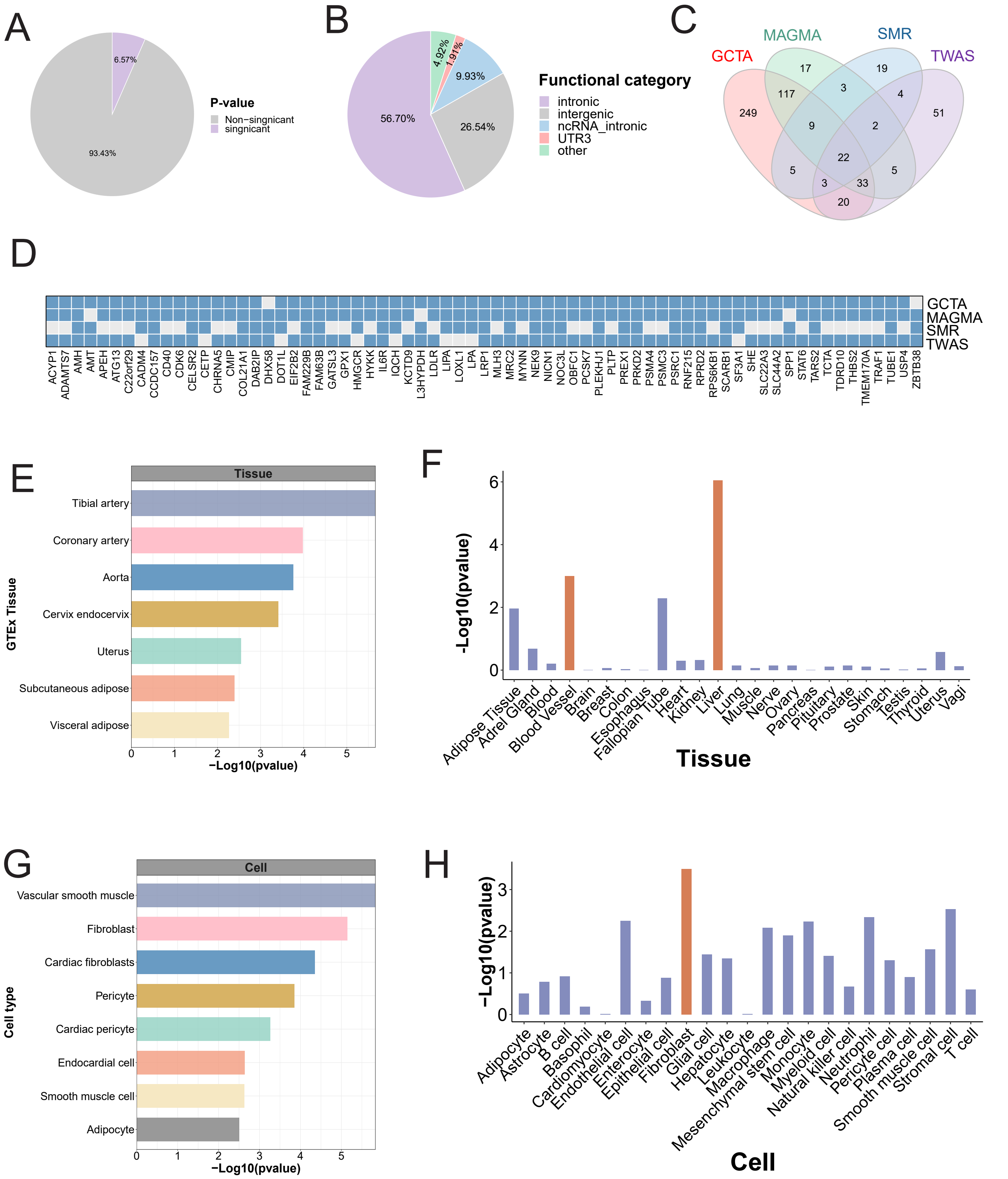

Supplementary Figure 9. Interpretation of AAA-associated variants identified by GWAS – variants, genes, tissues and cell types. A. Distribution of GWAS P values. B. Genic locations of the significant SNVs (GWAS P < 5 × 10-8). C. AAA-related genes identified by four gene analysis methods. D. AAA-related genes identified by minimally three of the four gene analysis methods. E. Tissue enrichment based on SNV heritability in tissue-specific genes defined in GTEx, computed by S-LDSC. F. Tissue enrichment based on tissue-specific genes defined in GTEx, computed by Tissue Specific Expression Analysis (TSEA). Orange represents significantly enriched tissues passing the Bonferroni corrected P value threshold. G. Cell type enrichment based on SNV heritability in cell type-specific enhancers from CATLAS, using S-LDSC. H. Cell type enrichment based on cell type-specific genes defined in various single-cell transcriptome studies, computed by Cell Specific Expression Analysis (CSEA). Orange represents significantly enriched cell types passing the Bonferroni corrected P value threshold.

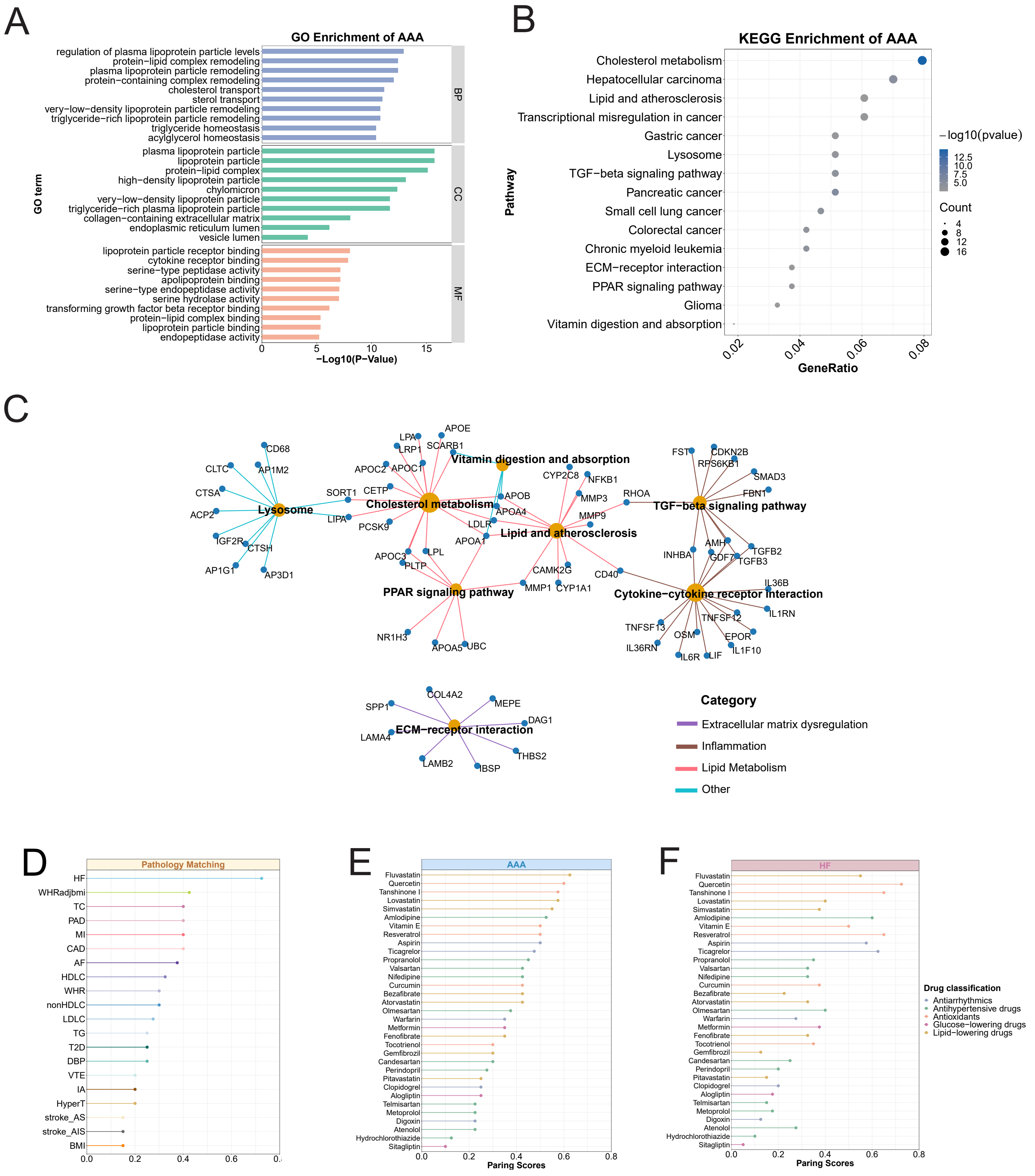

Supplementary Figure 10. Interpretation of AAA-associated variants identified by GWAS – Biological pathways and drugs. A. GO enrichment analyses based on the genes identified by any of the four gene analysis methods. B. KEGG enrichment analyses based on the genes identified by any of the four gene analysis methods. C. Interactions among the genes (blue circles) from the 8 enriched pathways (orange circles) in KEGG enrichment analysis. The colors of the lines represent different functional categories. D. Matching scores between pathological pathways of AAA and CMTs. E. Matching scores between AAA pathological pathways and drug pharmacological pathways. F. Matching scores for biological pathways in heart failure and the drug pharmacological pathways.

Supplementary Table 1. Horizontal pleiotropy analyses between AAA and cardiometabolic traits by MR-Egger.

Exposure	Outcome	Egger intercept	SE	*P* value
AAA	CAD	-0.0016	0.0052	0.77
AAA	MI	0.0014	0.0072	0.85
BMI	AAA	-0.0027	0.0023	0.25
CAD	AAA	-0.0002	0.0052	0.97
DBP	AAA	-0.0042	0.0031	0.17
HDLC	AAA	-0.0022	0.0011	0.04
HyperT	AAA	0.0019	0.0053	0.71
LDLC	AAA	-0.0013	0.0016	0.41
nonHDLC	AAA	-0.0023	0.0016	0.14
PP	AAA	0.0041	0.0029	0.16
TC	AAA	-0.0020	0.0014	0.16
TG	AAA	0.0002	0.0012	0.90
