# Supplementary material for: Abdominal aortic aneurysm and cardiometabolic traits share strong genetic susceptibility to lipid metabolism and inflammation
Source: Nat Commun. 2024 Jul 5;15:5652. doi: 10.1038/s41467-024-49921-7 (PMC11226445; doi:10.1038/s41467-024-49921-7)
Supplement: Supplementary file 3 — Description of Additional Supplementary Files [file 41467_2024_49921_MOESM3_ESM.pdf]

Description of Additional Supplementary Files

Supplementary Data 1. Summary of GWAS data.

Supplementary Data 2. Cross-trait meta-analysis between AAA and cardiometabolic traits.

Supplementary Data 3. Colocalization analysis of sentinel SNPs between AAA and cardiometabolic traits.

Supplementary Data 4. The list of AAA-trait pairs-related genes identified by four gene-based analyses.

Supplementary Data 5. Mechanism and indication for 33 drug candidates.

Supplementary Data 6. Clinical trials related to 33 drug candidates.
